# Irregular rhythm?

**DOI:** 10.1007/s12471-019-1230-9

**Published:** 2019-01-28

**Authors:** A. W. G. J. Oomen, F. F. J. Rozestraten

**Affiliations:** 1 Department of Cardiology, Amphia Ziekenhuis, Breda, The Netherlands; 2 Department of Cardiology, Catharina Ziekenhuis, Eindhoven, The Netherlands

An 84-year-old female presented with palpitations. Her cardiac medical history revealed persistent atrial fibrillation. The findings of a physical examination were normal. An electrocardiogram was recorded (Fig. 1). What is the rhythm and what is the mechanism for the varying conduction?

**Fig. 1:**
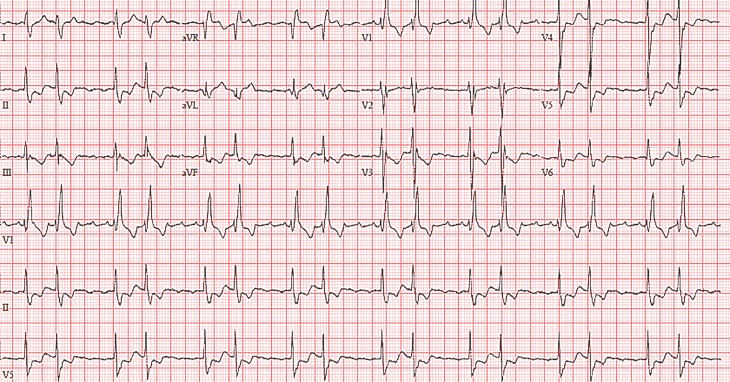
Electrocardiogram at presentation

## Answer

You will find the answer elsewhere in this issue.

